# Fetal, Infant and Maternal Outcomes among Women with Prolapsed Membranes Admitted before 29 Weeks Gestation

**DOI:** 10.1371/journal.pone.0168285

**Published:** 2016-12-21

**Authors:** Julie E. Robertson, Sarka Lisonkova, Tang Lee, Dane A. De Silva, Peter von Dadelszen, Anne R. Synnes, K. S. Joseph, Robert M. Liston, Laura A. Magee, for the Canadian Perinatal Network and Canadian Neonatal Network Collaborative Groups

**Affiliations:** 1 Department of Obstetrics and Gynaecology, University of British Columbia and the British Columbia’s Children’s and Women’s Hospital and Health Centre, Vancouver, British Columbia, Canada; 2 School of Population and Public Health, University of British Columbia, Vancouver, British Columbia, Canada; 3 Department of Pediatrics, University of British Columbia and the British Columbia’s Children’s and Women’s Hospital and Health Centre, Vancouver, British Columbia, Canada; Centre Hospitalier Universitaire Vaudois, FRANCE

## Abstract

**Background:**

Few studies have examined fetal, infant and maternal mortality and morbidity among pregnant women at very early gestation with an open cervix and prolapsed membranes. We carried out a study describing the outcomes of women hospitalized with prolapsed membranes at 22–28 weeks’ gestation.

**Methods:**

We prospectively recruited women with singleton pregnancies admitted at 22–28 weeks’ gestation to tertiary hospitals of the Canadian Perinatal Network between 2005 and 2009. Time-to-delivery, perinatal death, neonatal intensive care unit (NICU) admission, severe neonatal morbidity and severe maternal morbidity were compared between women admitted at 22–25 vs. 26–28 weeks gestation. Logistic regression was used to estimate adjusted odds ratios (AOR) and 95% confidence intervals.

**Results:**

129 women at 22–25 weeks gestation and 65 women at 26–28 weeks gestation were admitted to hospital and the median time-to-delivery was 4 days in both groups. Stillbirth rates were 12.4% vs 4.6% among women admitted at earlier vs later gestation (AOR 2.8, 95% CI 0.5–14.8), while perinatal death rates were 38.0% vs 6.1% (AOR 14.1, 95% CI 3.5–59.0), respectively. There were no significant differences in NICU admission and severe morbidity among live-born infants; 89.4% and 82.3% died or were admitted to NICU, (P value 0.18), and 53.9% vs 44.0% of NICU infants had severe neonatal morbidity (P value 0.28). Antibiotics, tocolysis and cerclage did not have a significant effect on perinatal death. Maternal death or severe maternal morbidity occurred in 8.5% and 6.2% of women admitted at 22–25 vs 26–28 weeks (AOR 1.2, 95% CI 0.4–4.2).

**Conclusion:**

Perinatal mortality among women with prolapsed membranes at very early gestation is high, although significantly lower among those admitted at a relatively later gestational age. Rates of adverse maternal outcomes are also high. This information can be used to counsel women with prolapsed membranes at 22 to 28 weeks gestation.

## Introduction

Preterm birth remains a major cause of neonatal morbidity and mortality, with especially high rates of death and illness at very early gestation. Survival rates are estimated to be approximately 54% at 25 weeks of gestation, 38% at 24 weeks, and 23% at 23 weeks gestation [1]. Despite advances in neonatal care, the morbidity for infants born between 24 and 26 weeks of gestation continues to be significant; nearly 50% of infants born before 25 completed weeks have one or more disability [2].

Studies show that a short cervix is an accurate predictor of preterm birth [3–5]; an open cervix with prolapsed membranes at midtrimester conveys a more serious prognosis. The incidence of short cervix ranges from about 2–6% and the incidence of an open cervix is likely lower [6–8]. Exposure of membranes to the vaginal flora may predispose to infection or rupture of membranes, and prolapsed membranes presage imminent delivery especially in the absence of intervention [9–11]. Despite the grave prognosis associated with prolapsed membranes at early gestation, fetal, infant and maternal mortality and morbidity among women diagnosed with an open cervix with prolapsed membranes have not been adequately studied. The few small clinical trials that have compared rescue cerclage with expectant management involved clinically heterogeneous populations; these trials included women with prolapsed membranes diagnosed at varying gestational ages (some <20 weeks gestation) [12–17].

We carried out a study describing fetal, infant and maternal outcomes among a large cohort of pregnant women with a diagnosis of prolapsed membranes prior to 29 weeks of gestation. We compared women with a diagnosis of prolapsed membranes at 22–25 weeks versus 26–28 weeks of gestation in terms of time to delivery, obstetric intervention, maternal morbidity, and birth outcomes. In addition, we examined the use of antenatal steroids, antibiotics, tocolysis and cerclage in relation to birth outcomes among these women.

## Methods and Materials

We carried out a prospective study of women admitted to hospital with prolapsed membranes at 22–28 weeks’ gestation between 2005 and 2009 using data collected prospectively by the Canadian Perinatal Network (CPN). During this period, CPN collected information on all women admitted at 22+0 to 28+6 weeks gestation for pregnancy complications to participating tertiary perinatal hospitals and perinatal centers across Canada. Information about maternal demographic, behavioral and clinical characteristics, pregnancy complications and birth outcomes was abstracted from hospital charts by trained data abstractors.

Enrolled women were followed up until delivery including during re-hospitalization. Information on stillbirth, maternal death, maternal morbidity and intensive care unit (ICU) admissions was abstracted from maternal hospital charts. While information about live born infants who did not require neonatal intensive care unit (NICU) admission was obtained directly from the neonatal hospital records, information on infants admitted to NICU was obtained though a linkage to the Canadian Neonatal Network (CNN), which collected detailed information on infants admitted to NICUs across Canada. Collected data included infant death before discharge and neonatal morbidity, e.g., bronchopulmonary dysplasia (BPD) and severe intraventricular hemorrhage (IVH). In addition, CNN collected data to calculate the Score for Neonatal Acute Physiology (SNAP-II). This score measures the severity of the neonatal condition after birth and within the first 24 hours, and includes, for example, urine output, neonatal blood pressure, neonatal seizures, and oxygen saturation. High SNAP II values indicate higher degree of neonatal morbidity and a high risk for neonatal death.

Our study included women with singleton pregnancies admitted for, or diagnosed with, prolapsed membranes during hospitalization at 22–28 weeks gestation. Pregnancies with congenital anomalies were excluded. Women with prolapsed membranes were defined as those with a dilated cervix with membranes at or beyond the external os as visualized on speculum examination, or those with any cervical dilatation of the external os by endovaginal ultrasound examination. Women with severe prolapsed membranes were defined as those with a ≥2cm dilated cervix or ‘hour-glassing’ (i.e., membranes beyond the cervical os).

Maternal outcomes of interest included time to delivery after diagnosis of prolapsed membranes (in days), maternal death and severe maternal morbidity. Severe maternal morbidity was defined as any of the following potentially life threatening conditions: abruptio placentae, need for injectable antihypertensives or positive inotropic support, myocardial ischemia/infarction, blindness, eclampsia, coma (Glasgow score<13), stroke, other adverse neurological events, disseminated intravascular coagulation, hysterectomy, embolization, sepsis, endometritis, intubation, non-invasive ventilation, pulmonary edema, oxygen requirement for >1 hour (>50% O_2_), acute renal failure, dialysis, hepatic failure/dysfunction, hepatic hematoma/rupture, and thromboembolism. Admission to an intensive care unit (ICU) or high dependency obstetrical unit and chorioamnionitis (suspected or confirmed) were also studied.

Fetal/neonatal outcomes of interest included fetal death, neonatal death before discharge, admission to the neonatal intensive care unit (NICU), and severe neonatal morbidity (for NICU infants only) defined as any of the following: BPD defined as need for supplemental oxygen or respiratory support at 36 weeks postmenstrual age, IVH grade 3 and 4, retinopathy of prematurity (ROP, stage 3 or higher), necrotizing enterocolitis (NEC), neonatal sepsis, neonatal seizures, and central nervous system shunt placement.

The study population was categorized into two subcohorts, women who were diagnosed with prolapsed membranes at 22–25 weeks and 26–28 weeks gestation. The statistical significance of differences in maternal and clinical characteristics between the groups was assessed using a chi-square or Fisher’s exact test, while differences in the time to delivery between the groups were assessed using a log-rank test. Maternal and birth outcomes were compared using logistic regression to adjust for potential confounders. For maternal outcomes, the confounders included in the logistic model were maternal age, marital status, gravidity, smoking during pregnancy, and alcohol and drug use. For birth outcomes, adjustment was also made for previous stillbirth, previous preterm birth <37 weeks, and use of cerclage, antenatal corticosteroids, antibiotics, or tocolysis. In the primary analysis we did not adjust for choriamnionitis, short cervix, antepartum hemorrhage, placental abruption, and preterm labour since these variables could be in the pathway between prolapsed membranes and perinatal outcomes. However, in secondary analyses of perinatal outcomes we additionally adjusted for these variables in order to ascertain any (indirect) effects potentially mediated by these variables.

Analyses were also carried out to assess the potential effects of cerclage, antepartum antibiotics, antenatal corticosteroids, and tocolysis on live birth ≥34 weeks, NICU admission, stillbirth and perinatal death. Interaction terms were added to the logistic regression model to assess whether the efficacy of these treatments was different among women with prolapsed membranes at 22–25 weeks vs 26–28 weeks gestation.

All analyses were carried out using SAS software, version 9.3 (SAS Institute Inc., Cary NC). The Research Ethics Committee at each participating centre reviewed and approved the CPN protocol as a quality assurance project and waived the requirement for individual informed consent. The names of the participating centres, the Research Ethics Committee and the ethics approval certificate number are as follows: Victoria General Hospital, Victoria, BC, Island Health Research Ethics Board, Certificate No. H2007-68; British Columbia Women’s Hospital and Health Centre, Vancouver, BC, University of British Columbia Research Ethics Board, Certificate No. H05-70359; Foothills Medical Centre, Calgary, AB, Conjoint Health Research Ethics Board, Certificate No. E-21281; Royal Alexandra Hospital, Edmonton, AB, Health Research Ethics Board, Certificate No. Pro00001472_REN3; Royal University Hospital, Saskatoon, SA, University of Saskatchewan Research Ethics Board, Certificate No. Bio 06–04; Regina General Hospital, Regina, SA, Ethics Committee, Regina Qu’Appelle Health Region, Certificate No. REB-06-33; Kingston General Hospital, Kingston, ON, Queen's University Health Sciences & Affiliated Teaching Hospital Research Ethics Board, Certificate No. OBGY-153-06; McMaster University Medical Centre, Hamilton, ON, McMaster Research Ethics Board, Certificate No. 09–452; London Health Sciences Centre, London, ON, Health Sciences Research Ethics Board—Western University, Certificate No. 5677; Mount Sinai Hospital, Toronto, ON, Mount Sinai Hospital Research Ethics Board, Certificate No. 06-0019-C; The Ottawa Hospital, Ottawa, ON, Ottawa Health Science Network Research Ethics Board, Certificate No. 2006145-01H; Universitaire de Sherbrooke, Sherbrooke, QU, CHUS Human Research Ethics Committee, Certificate No. 06–128; Centre Hôspitalier de L’Université Laval, Quebec City, QU, Comité d'éthique de la recherché, Certificate No. 95.05.10; Centre Hôspitalier Universitaire Sainte-Justine, Montreal, QU, Research Ethics Board of the Sainte-Justine University Hospital, Certificate No. 2275; IWK Health Centre, Halifax, NS, IWK Health Centre Research Ethics Board, Certificate No. 1004849; and the Women’s Health Program, Eastern Health, St. John’s, NFL, Health Research Ethics Authority, Certificate No. 06.51.

## Results

The study population consisted of 194 pregnant women with singleton non-anomalous fetuses who were diagnosed with or admitted to tertiary care hospitals or perinatal centers in Canada for prolapsed membranes at 22–28 week gestation between 2005 and 2009. 129 women had a diagnosis of prolapsed membranes between 22 and 25 weeks, while 65 women were diagnosed between 26 and 28 weeks gestation. Women in the latter group were more likely to be unmarried (18.5% vs 8.5%, Table 1), but did not differ significantly with regard to primigravidity, obstetric history, smoking in pregnancy and other factors.

**Table 1:** Characteristics of women with prolapsed membranes at 22–25 weeks vs 26–28 weeks gestation (singleton pregnancies without congenital anomalies).

Demographic and clinical factors	Prolapsed membranes		P-value
	22–25 weeks	26–28 weeks	
	N = 129 (%)	N = 65 (%)	
Maternal age (years) <20	5 (3.9)	4 (6.1)	0.38
20–24	27 (20.9)	11 (16.9)	
25–29	42(32.6)	17 (26.1)	
30–34	30 (23.3)	23 (35.4)	
35–39	18 (13.9)	9 (13.8)	
≥40	7 (5.4)	1 (1.5)	
Unmarried	11 (8.5)	12 (18.5)	0.04
Primigravida	42 (32.6)	25 (38.5)	0.41
Multipara	58 (45.0)	27 (41.5)	0.93
Prior stillbirth	7 (5.4)	2 (3.1)	0.72
Prior birth <34 weeks	19 (14.7)	6 (9.2)	0.28
Prior birth <37 weeks	27 (20.9)	11 (16.9)	0.51
Smoking during pregnancy	31 (24.4)	19 (29.7)	0.43
Alcohol	13 (10.2)	3 (4.8)	0.27
Drugs	4 (3.2)	6 (9.4)	0.09
Male fetus/infant	65 (50.4)	27 (41.5)	0.24
Gestational age at diagnosis: No. (%)			<0.001
22 weeks	16 (12.4)	-	
23 weeks	38 (29.5)	-	
24 weeks	46 (35.7)	-	
25 weeks	29 (22.5)	-	
26 weeks	-	24 (36.9)	
27 weeks	-	18 (27.7)	
28 weeks	-	23 (35.4)	
Time-to-delivery (days)*			
Median (IQR)	4 (17)	4 (13)	0.47
No. (%) 0 days	13 (10.1)	13 (20.0)	0.40
1 day	19 (14.7)	8 (12.3)	
2 days	13 (10.1)	5 (7.7)	
3–5 days	32 (24.8)	12(18.4) (18.5)	
6–10 days	11 (8.5)	8 (12.3)	
>10 days	41 (31.8)	19 (29.2)	
Gestational age at delivery (weeks)			
Median (IQR)	25 (2)	28 (2)	<0.001
No. (%) 22–25 weeks	94 (72.9)	0 (0)	<0.001
26–28 weeks	17 (13.2)	46 (70.8)	
29–33 weeks	6 (4.6)	9 (13.8)	
34–36 weeks	4 (3.1)	5 (7.7)	
37–43 weeks	8 (6.2)	5 (7.7)	

* Number of days between diagnosis of prolapsed membranes and delivery.

Note: There were 3 missing values for smoking during pregnancy, and 4 missing values for alcohol and drug use.

**Fig 1:**
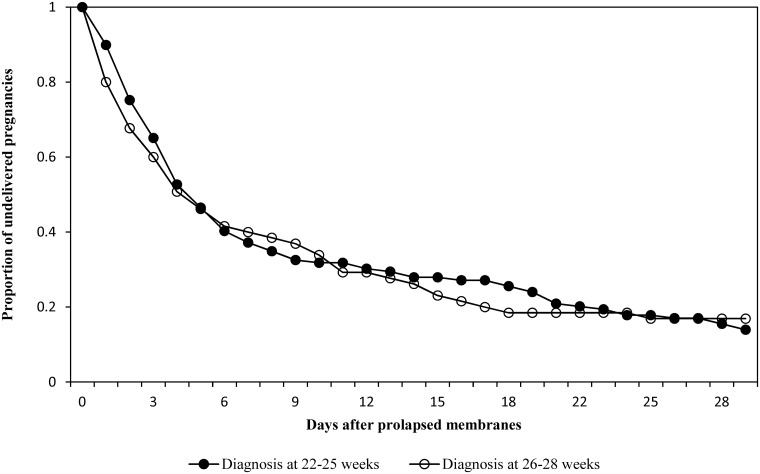
Proportion of undelivered pregnancies after a diagnosis of prolapsed membranes at 22–25 vs 26–28 weeks’ gestation among women with non-anomalous singleton pregnancies.

Although those diagnosed at earlier gestation tended to deliver at earlier gestation, the median number of days to delivery was 4 days in both cohorts. Approximately 25% vs 32% of women delivered within one day after diagnosis in the 22–25 weeks vs 26–28 weeks cohorts respectively, while 32% vs 29% delivered more than 10 days after diagnosis (Table 1). The duration between diagnosis and delivery did not differ significantly between the groups (Fig 1). Among women with prolapsed membranes diagnosed at 26–28 weeks, all stillbirths occurred within the first 2 days after diagnosis (3 stillbirths), while among women with an earlier diagnosis, the majority of stillbirths occurred after 2 days following diagnosis of prolapsed membranes (11 out of 16 stillbirths).

There were no significant differences in pregnancy complications between women diagnosed at 22–25 weeks vs 26–28 weeks, with both groups having similar rates of preterm prelabour rupture of membranes, preterm labour, short cervix, antepartum hemorrhage, placenta accreta, placental abruption, gestational diabetes, hypertension in pregnancy, and cesarean delivery (Table 2). The rates of chorioamnionitis were also similar (9.3% vs 10.8%, respectively). However, women with an earlier diagnosis of prolapsed membranes were significantly more likely to receive antibiotics (73.6% vs 58.5%), and cerclage (23.3% vs 4.6%), but less likely to receive antenatal corticosteroids (72.9% vs 86.5%) compared with women diagnosed at 26–28 weeks (Table 2).

**Table 2:** Pregnancy complications, labour characteristics and interventions among women with prolapsed membranes at 22–25 vs 26–28 weeks gestation (singleton pregnancies without congenital anomalies).

Pregnancy complications/interventions	Prolapsed membranes		P-value
	22–25 weeks	26–28 weeks	
	N = 129 (%)	N = 65 (%)	
Complications			
Severe prolapse of membranes	96 (74.4)	42 (64.6)	0.16
PPROM	21 (16.3)	9 (13.8)	0.66
Preterm labour	34 (26.4)	19 (29.2)	0.67
Short cervix	27 (20.9)	15 (23.1)	0.73
Antepartum hemorrhage	34 (26.4)	13 (20.0)	0.33
Placenta accreta	0 (0.0)	1 (1.5)	1.00
Abruptio placentae	9 (7.0)	2 (3.1)	0.27
Chorioamnionitis	12 (9.3)	7 (10.8)	0.75
Sepsis	2 (1.6)	0 (0.0)	0.55
Gestational diabetes	1 (0.8)	1 (1.5)	1.00
Hypertension in pregnancy	1 (0.8)	1 (1.5)	1.00
Labour and intervention			
Spontaneous labour	97 (75.2)	49 (75.4)	0.98
Induction of labour	14 (10.8)	5 (7.7)	0.48
Augmentation of labour	8 (6.2)	4 (6.1)	1.00
Cesarean delivery	36 (27.9)	24 (36.9)	0.20
Emergency*	31 (86.1)	23 (95.8)	0.22
Vaginal delivery	93 (72.1)	41 (63.8)	
Assisted*	10 (10.7)	3 (7.3)	0.75
Antenatal steroids	94 (72.9)	56 (86.5)	0.04
Antibiotics (antepartum)	95 (73.6)	38 (58.5)	0.03
Tocolysis	63 (48.8)	28 (43.1)	0.45
Cerclage	30 (23.3)	3 (4.6)	0.001

PPROM denotes preterm prelabour rupture of membranes.

* Emergency cesarean rate calculated among women with cesarean deliveries and assisted vaginal delivery rates calculated among women with vaginal deliveries.

Women in both groups had relatively high rates of maternal morbidity, and no significant differences between groups were observed. There was 1 maternal death among women with prolapsed membranes at 22–25 weeks, and 8.5% vs 6.2% of women with prolapsed membranes at 22–25 vs 26–28 weeks gestation experienced death or maternal morbidity (Table 3).

**Table 3:** Maternal death and severe maternal morbidity rates among women with prolapsed membranes at 22–25 vs 26–28 weeks gestation (singleton pregnancies without congenital anomalies).

Maternal death/morbidity	Prolapsed membranes		P-value	Odds ratio	Adjusted odds ratio
	22–25 weeks	26–28 weeks		(95% CI)	(95% CI)
	N = 129 (%)	N = 65 (%)		(22–25 vs 26–28 wks)	(22–25 vs 26–28 wks)
Maternal death	1 (0.78)	0 (0.00)	1.00	-	-
Intensive Care Unit admission	3 (2.33)	3 (4.62)	0.40	0.49 (0.10–2.51)	0.51 (0.10–2.65)
Blood transfusion/blood products	3 (2.33)	0 (0.00)	0.55	-	-
Severe maternal morbidity*	8 (6.20)	2 (3.08)	0.50	2.08 (0.43–10.1)	2.17 (0.41–11.47)
Maternal death/morbidity†	11 (8.53)	4 (6.15)	0.78	1.42 (0.43–4.65)	1.23 (0.36–4.19)

Adjusted odds ratio adjusted for maternal age, marital status, gravidity, smoking during pregnancy, alcohol and drug use.

* Severe maternal morbidity included abruptio placentae, need for injectable antihypertensives, positive inotropic support, myocardial ischemia/infarction, blindness, eclampsia, coma (Glasgow score<13), stroke, adverse neurological events, disseminated intravascular coagulation, hysterectomy, embolization, sepsis, endometritis, intubation, non-invasive ventilation, pulmonary edema, oxygen requirement for >1 h (>50% O2), acute renal failure, dialysis, hepatic failure/dysfunction, hepatic hematoma/rupture, and thromboembolism.

^†^ Maternal death/morbidity included maternal death, severe maternal morbidity, blood transfusion/blood products, or Intensive Care Unit admission.

The perinatal mortality rate was significantly higher among women with prolapsed membranes diagnosed at 22–25 vs 26–28 weeks (38.0 vs 6.1 per 100 total births; odds ratio 9.3, 95% CI 3.2–27.3; Table 4). The strength of this association was essentially unchanged after adjustment for maternal characteristics and treatments received (adjusted odds ratio AOR 9.1, 95% CI 2.5–33.5). Adjustment for variables potentially in the causal pathway (chorioamnionitis, short cervix, etc) increased the strength of the association and also the width of the 95% confidence intervals (AOR 14.1, 95% CI 3.5–59.0). Stillbirth rates were higher among women with early vs later diagnosis, but these differences were not statistically significant (12.4 vs 4.6 per 100 total births, P value 0.12). The majority of stillbirths occurred intra-partum (87% and 67% in each group). A lower proportion of live born infants was admitted to NICU among women with prolapsed membranes at 22–25 vs 26–28 weeks (68.1% vs 82.3%, P value 0.04). However, the rates of delivery ≥34 weeks were similar in the 2 groups, while rates of death before hospital discharge were significantly higher among women with prolapsed membranes at 22–25 weeks (Table 4).

**Table 4:** Perinatal outcomes among women with prolapsed membranes at 22–25 vs 26–28 weeks gestation (singleton pregnancies without congenital anomalies).

Perinatal outcomes	Prolapsed membranes		P-value	Odds ratio	Adjusted odds	Adjusted odds
				(95% CI)	ratio^1^ (95% CI)	ratio^2^ (95% CI)
	22–25 weeks	26–28 weeks				
	N = 129 (%)	N = 65 (%)				
Perinatal death*†	49 (38.0)	4 (6.1)	<0.001	9.34 (3.20–27.3)	9.08 (2.46–33.5)	14.1 (3.50–59.0)
Stillbirth	16 (12.4)	3 (4.6)	0.12	2.93 (0.82–10.4)	2.08 (0.44–9.78)	2.77 (0.52–14.8)
Live birth	N = 113	N = 62				
Gestational age at delivery >33 weeks‡	12 (10.6)	10 (16.1)	0.29	0.62 (0.25–1.53)	0.74 (0.22–2.46)	0.72 (0.23–2.30)
Death before discharge*	33 (29.5)	1 (1.6)	<0.001	25.1 (3.33–188.4)	22.9 (2.69–194.8)	25.5 (3.00–217.1)
NICU admission	77 (68.1)	51 (82.3)	0.04	0.46 (0.22–0.99)	0.38 (0.15–0.96)	0.40 (0.15–1.06)
Death before discharge/NICU admission	101 (89.4)	51 (82.3)	0.18	1.82 (0.75–4.40)	1.63 (0.57–4.67)	1.69 (0.53–5.39)

* One infant in each group had missing data on NICU follow-up.

^†^ Includes stillbirth or death before hospital discharge.

^‡^ No perinatal death occurred after 33 weeks gestation.

Adjusted odds ratio^1^: Adjusted for maternal age, marital status, gravidity, smoking during pregnancy, alcohol and drug use, previous stillbirth, previous preterm birth less than 37 weeks, and use of antepartum antibiotics, cerclage, tocolysis and steroid.

Adjusted odds ratio^2^: Additionally adjusted for choriamnionitis, short cervix, antepartum hemorrhage, placental abruption, and preterm labour.

NICU infants born to mothers diagnosed at 22–25 weeks gestation had a higher SNAP-II score and a higher number of days on ventilation (S1 Table). A larger proportion of the NICU infants of mothers with prolapsed membranes at 22–25 weeks stayed in the NICU for more than 90 days as compared with NICU infants born to mothers with prolapsed membranes at 26–28 weeks gestation (31.6% vs 8.0%, P value 0.002). However, the combined outcome of death or severe neonatal morbidity was not significantly different between NICU infants born to mothers diagnosed at 22–25 vs 26–28 weeks (59.2% vs 44.0%, respectively, P value 0.09).

Treatment with antibiotics, tocolysis and cerclage did not have a significant effect on perinatal death (Table 5). However, antenatal corticosteroid use was associated with a lower risk of stillbirth (AOR 0.14, 95% CI 0.02–0.98) and perinatal death (AOR 0.21, 95% CI 0.06–0.70). Interaction terms showed that the observed effects of these interventions were not significantly different between women with prolapsed membranes at 22–25 weeks vs women with prolapsed membranes at 26–28 weeks gestation except for the association between antepartum antibiotics and live birth ≥34 weeks. Antepartum antibiotics were non-significantly positively associated with live birth ≥34 weeks among women with prolapsed membranes at 22–25 weeks (AOR 4.41, P value 0.25), while a significant negative association was found among women with prolapsed membranes at 26–28 weeks (AOR 0.01, P value 0.01).

**Table 5:** Adjusted odds ratios expressing the association between antibiotics, antenatal steroids, cerclage and tocolysis on perinatal outcomes among women with prolapsed membranes 22–28 weeks gestation (singleton pregnancies without congenital anomalies).

Birth outcome	Adjusted odds ratios* (95% CI) for effects of			
	Antibiotics	Antenatal steroids	Cerclage	Tocolysis
Perinatal death†	1.54 (0.49–4.87)	0.21 (0.06–0.70)	0.21 (0.05–0.85)	0.75 (0.28–2.03)
Stillbirth	1.15 (0.17–7.75)	0.14 (0.02–0.98)	1.23 (0.15–10.07)	1.58 (0.26–9.77)
Death before discharge	2.52 (0.48–13.36)	0.27 (0.05–1.46)	0.17 (0.02–1.18)	0.75 (0.18–3.10)
Death before discharge or NICU admission	1.52 (0.50–4.57)	0.13 (0.01–1.30)	0.68 (0.19–2.48)	1.22 (0.45–3.30)
Delivery >33 weeks		5.94 (0.52–16.8)	3.08 (0.56–16.84)	1.00 (0.23–4.28)
Prolapsed membranes at 22–25 weeks	4.41 (0.36–54.7)			
Prolapsed membranes at 26–28 weeks	0.01 (0.00–0.40)			

* Adjusted for gestational age at the diagnosis of prolapsed membranes, maternal age, marital status, gravidity, smoking during pregnancy, alcohol and drug use, previous stillbirth, previous preterm birth less than 37 weeks, and other co-interventions included in the Table. Adjusted odds ratio for the association between antibiotics use and delivery of a live newborn at >33 weeks differed between women diagnosed with prolapsed membranes at 22–25 and 26–28 weeks gestation.

^†^ Includes stillbirth or death before hospital discharge.

## Discussion

Our study showed that the likelihood of a pregnancy continuing for any significant duration following a diagnosis of prolapsed membranes was low with a median duration of pregnancy after the diagnosis of prolapsed membranes of 4 days. Women with prolapsed membranes had a relatively high rate of other pregnancy complications, such as preterm prelabour rupture of membranes, preterm labour, antepartum hemorrhage, chorioamnionitis and placental abruption. Similarly, women with prolapsed membranes had a high rate of severe maternal morbidity, regardless of gestational age at diagnosis. Perinatal mortality rates were higher among women diagnosed with prolapsed membranes at 22–25 weeks compared with women diagnosed at 26–28 weeks gestation due to a higher stillbirth rate and higher rates of infant death before discharge.

A few previous studies on women with prolapsed membranes have reported a 2 to 3 week latency between the diagnosis of prolapsed membranes and delivery [12–17]. These studies compared women who received emergency cerclage, and women who had bed rest or were managed without intervention. The studies were typically small, although one study included 161 women between 17 and 26 weeks gestation and reported a median time-to-delivery interval of 3 days [16].

Perinatal mortality rates in our study were high, especially among mothers with prolapsed membranes diagnosed at 22–25 weeks gestation (38.0%); these high rates are consistent with the findings from previous studies [13–16]. Severe maternal morbidity rates among women with prolapsed membranes at 22 to 28 weeks gestation were also high; 8.5% of those diagnosed at 22–25 weeks and 6.2% of those diagnosed at 26–28 weeks experienced maternal death or severe morbidity. Few previous studies have examined maternal morbidity related to a diagnosis of prolapsed membranes in early gestation and these were restricted to reports of sepsis and chorioamnionitis [12,13,17].

The results of our study are important for counseling patients. The probability of neonatal survival without major morbidity is low when a diagnosis of prolapsed membranes is made at 22–25 weeks. Expectant management in this situation may be an option, but is associated with poor fetal and neonatal outcomes and significant maternal risk. Conversely, when a diagnosis of prolapsed membranes is made at 26–28 weeks of gestation, perinatal mortality rates are relatively lower; therefore, patients can be reassured that expectant management (without cerclage) is a reasonable option and results in relatively low rates of neonatal morbidity and mortality.

In our study, tocolysis or cerclage did not appear to offer any benefit to women with prolapsed membranes at 22 and 28 weeks. However, the non-experimental nature of our study precludes strong inferences because of the potential for confounding by indication [18]. On the other hand, significant ‘protective’ associations were observed between antenatal corticosteroids use and stillbirth and perinatal death. In addition, antepartum antibiotics appeared to have a different effect in prolonging gestation for women at 22–25 weeks vs women at 26–28 weeks at diagnosis of prolapsed membranes; antibiotics were protective among women at 22–25 weeks and a risk factor at 26–28 weeks. These associations, however, were possibly biased by reverse causality and confounded by indication; e.g., women with a stillbirth or at imminent risk of stillbirth were likely not offered antenatal corticosteroid prophylaxis and antibiotics may have been preferentially given to women with signs of infection especially at 26–28 week.

The strengths of our study include its relatively large size, the inclusion of data from multiple tertiary centres in Canada, and the reporting of both neonatal and maternal outcomes. Our results were unaffected by differences in medical insurance availability, as all Canadian residents are covered by universal health care insurance. Our study has a few limitations. First, we did not have information on the intention to continue pregnancy among women with prolapsed membranes at 22–25 weeks. It is possible that some women did not wish to continue pregnancy given the anticipated complications. Second, we did not know the exact timing of cerclage. In some cases, cerclage may have been placed and possibly removed prior to diagnosis of prolapsed membranes. Finally, we did not provide comparisons with pregnancy outcomes among women without prolapsed membranes, who mostly deliver at term gestation and have much lower rates of adverse maternal and neonatal outcomes.

## Conclusion

In conclusion, the results of this relatively large, multicentre study shows that diagnosis of prolapsed membranes between 22–25 weeks carries a high risk of perinatal morbidity and mortality. Relatively high rates of maternal morbidity are also to be expected in this population. Among women with prolapsed membranes at 26–28 weeks, neonatal morbidity and mortality are much lower, though maternal morbidity remains significant. Regardless of gestational age at diagnosis, fifty percent of women with prolapsed membranes at 22–28 weeks deliver within 4 days and only 10–15% of these women will deliver at 34 weeks of gestation or later. The prognostic information in our study, which shows differences in perinatal outcomes by gestational age at diagnosis of prolapsed membranes, will be useful for counseling pregnant women with prolapsed membranes at early gestation.

## Supporting Information

S1 Table Neonatal mortality and perinatal outcomes among NICU admitted infants of women with prolapsed membranes at 22–25 vs 26–28 weeks gestation (singleton pregnancies without congenital anomalies). (DOCX)
