# Supplementary material for: Fetal, Infant and Maternal Outcomes among Women with Prolapsed Membranes Admitted before 29 Weeks Gestation
Source: PLoS One. 2016 Dec 21;11(12):e0168285. doi: 10.1371/journal.pone.0168285 (PMC5176283; doi:10.1371/journal.pone.0168285)
Supplement: S1 Table — (DOCX) [file pone.0168285.s001.docx]

S1 Table. Neonatal mortality and perinatal outcomes among NICU admitted infants of women

with prolapsed membranes at 22-25 vs 26-28 weeks gestation (singleton pregnancies without congenital anomalies).

| Morbidity/mortality | Prolapsed membranes | | P-value |
| --- | --- | --- | --- |
|  | 22-25 weeks | 26-28 weeks |  |
|  | N=76* (%) | N=50* (%) |  |
| Gestational age in weeks (median, IQR) | 25 (2) | 28 (1) | <0.001 |
| Death in NICU | 9 (11.8) | 1 (2.0) | 0.10 |
| SNAPII on day 1 (median, IQR) | 19 (21) | 10 (11) | 0.001 |
| NICU length of stay > 1 day | 71 (93.4) | 50 (100) | 0.16 |
| > 7 days | 64 (84.2) | 49 (98.0) | 0.02 |
| > 30 days | 52 (68.4) | 40 (80.0) | 0.15 |
| > 90 days | 24 (31.6) | 4 (8.0) | 0.002 |
| Assisted ventilation for 1 day or more | 62 (83.8) | 39 (78.0) | 0.42 |
| Days on ventilation (median, IQR) | 25 (34) | 3 (14) | <0.001 |
| Severe neonatal morbidity† | 41 (53.9) | 22 (44.0) | 0.28 |
| Death/severe neonatal morbidity | 45 (59.2) | 22 (44.0) | 0.09 |
| * One infant in each group had missing data on NICU follow-up. | | |  |
| † Includes intraventricular hemorrhage (stage 3 or higher), retinopathy of prematurity  (grade 3 or higher), bronchopulmonary dysplasia, hypoxic ischemic encephalopathy,  necrotizing enterocolitis, central nervous system shunt placement, and extracorporeal  membrane oxygenation use. | | | |
| NICU denotes neonatal intensive care unit, SNAPPE denotes Score for Neonatal  Acute Physiology - Perinatal Extension. Median days on assisted ventilation calculated among infants who needed assisted ventilation only. IQR denotes interquartile range. | | | |
